# Efficacy of rintatolimod in the treatment of chronic fatigue syndrome/myalgic encephalomyelitis (CFS/ME)

**DOI:** 10.1586/17512433.2016.1172960

**Published:** 2016-05-25

**Authors:** William M Mitchell

**Affiliations:** ^a^ Department of Pathology, Microbiology & Immunology, Vanderbilt University, Nashville, USA

**Keywords:** Rintatolimod, chronic fatigue/myalgic encephalomyelitis, TLR3 agonist, clinical trials, Ampligen, dsRNA, clinical efficacy, clinical safety, primate/non-primate disassociation of toxicity

## Abstract

Chronic fatigue syndrome/ Myalgic encephalomyelitis (CFS/ME) is a poorly understood seriously debilitating disorder in which disabling fatigue is an universal symptom in combination with a variety of variable symptoms. The only drug in advanced clinical development is rintatolimod, a mismatched double stranded polymer of RNA (dsRNA). Rintatolimod is a restricted Toll-Like Receptor 3 (TLR3) agonist lacking activation of other primary cellular inducers of innate immunity (e.g.- cytosolic helicases). Rintatolimod also activates interferon induced proteins that require dsRNA for activity (e.g.- 2ʹ-5ʹ adenylate synthetase, protein kinase R). Rintatolimod has achieved statistically significant improvements in primary endpoints in Phase II and Phase III double-blind, randomized, placebo-controlled clinical trials with a generally well tolerated safety profile and supported by open-label trials in the United States and Europe. The chemistry, mechanism of action, clinical trial data, and current regulatory status of rintatolimod for CFS/ME including current evidence for etiology of the syndrome are reviewed.

## Introduction

Chronic fatigue syndrome/Myalgic encephalomyelitis (CFS/ME) is a seriously debilitating disorder in which disabling fatigue is a universal symptom in combination with a variety of variable symptoms that include sleep disorders, cognitive defects, joint/muscle pain, and headaches [1]. The fatigue is not improved by bed rest and may be worsened by physical activity. CFS/ME is an economically devastating illness whose societal costs to the U.S. economy are conservatively estimated at more than $20B yearly [2]. Heart failure, cancer, and suicide are the most common causes of death (59.6%) in patients with CFS/ME memorialized by the National CFIDS Foundation [3]. The mean age of death from cancer was 47.8 years and suicide was 39.3 years, which is significantly younger than the general population. Although limited by the informal collection of data, the analysis remains the most comprehensive to date and suggests a significantly increased risk of early death. The etiologic/pathogenic basis for CFS/ ME is unknown and may be multifactoral with a variety of microbes, hormonal, and immunological abnormalities linked to its pathogenesis [4–6]. Moreover, CFS/ME may have a familial component [7] and be dependent on genetic signatures [8–10]. Recently discovered time-dependent plasma immune signatures indicate a dynamic and evolving pathogenesis [11]. With no approved drug therapy available, treatment is aimed at symptom relief and improved ambulatory function [12]. These include over-the-counter and off-label prescription drugs, behavioral modifications, and graded exercise therapies.

The rationale for the initial open-label trials with rintatolimod in CFS/ME was based on its recognized broad antiviral and immunomodulatory properties as an inducer of interferon (IFN) [13]. These properties now are known to be mediated by its activity as a dsRNA toll-like receptor 3 (TLR3) agonist [14] in the induction of innate immunity and the initial cellular orchestration for the progression to adaptive immune responses, which are mediated in part by inflammatory chemokines and cytokines [15]. TLR3 is abundant in functional dendritic cells (DCs), central in the host adaptive immune response system [16]. All of the TLRs use a MyD88-dependent signaling pathway with the exception of TLR3 that uses the MyD88 independent TRIF pathway [17]. Two other dsRNA-activated inducers of gene expression that initiate innate immune responses are the cytosolic helicases mda5 and RIG-1. Rintatolimod, however, does not activate these helicases [18,19]. The selectivity of rintatolimod for TLR3 without helicase activation preserves the non-MyD88 TRIF pathway with its reduction in inflammatory cytokine induction observed with the MyD88/MAV pathways and is responsible at least in part for its improved safety record in clinical trials, compared to other forms of dsRNA that cause helicase activation [20]. The initial success of open-label trials provided the basis for the double-blind, placebo-controlled Phase II and Phase III clinical trials as well as its FDA designation as an Orphan Drug and a FDA-authorized treatment protocol.

## Overview of the CFS/ME clinical landscape

Public awareness of CFS/ME was initially trivialized from media coverage as ‘Yuppie Flu’ with an epicenter in Lake Tahoe, California, in the mid-1980s [21]. CFS/ME is recognized by the Federal government (CDC/FDA/NIH) as a disease syndrome that may be severely debilitating. Clinical trials are difficult to conduct due to spontaneous remissions although remissions become less frequent with increasing time from initial symptom onset. There have been several attempts by big pharma to introduce new pharmaceutical entities although none have been successful to date. Despite efforts to stimulate drug discovery for CFS/ME by the FDA [22], there appear to be no pending programs.

The majority of CFS/ME patients experience a sudden onset of symptoms, which many can identify as a specific date of acute onset [23,24]. The distinction of acute versus slow onset may represent a key element of differential pathogenesis, which has not been explored adequately. The signs and symptoms of acute onset are suggestive of an infectious basis in which some patients experience a prolonged extension of symptoms with profound fatigue as the unifying element.

## Introduction to the drug

**Figure 1.**
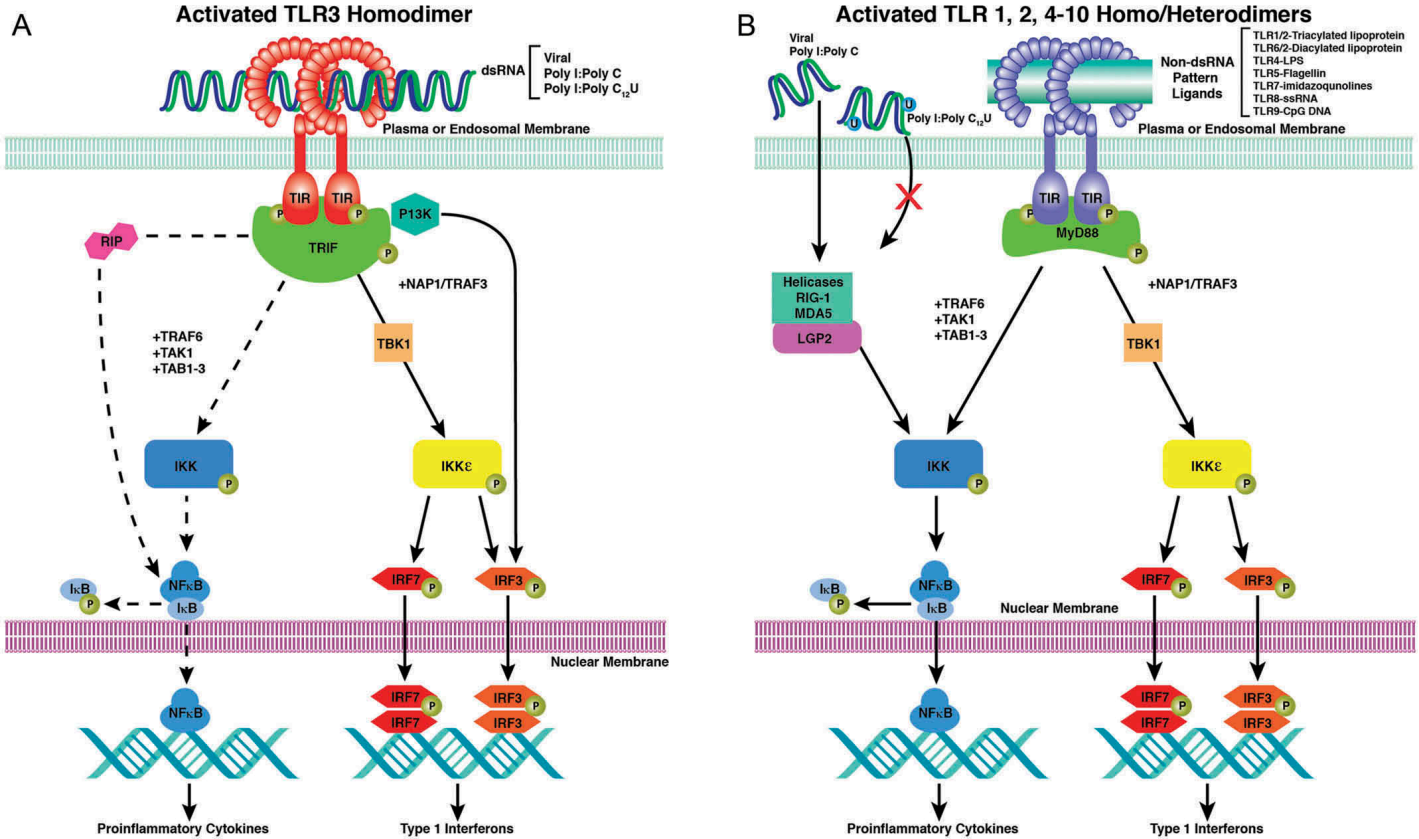
MyD88 dependent and Myd88 independent signaling pathways for the TLRs and helicases. A. Intracellular pathways for MyD88 independent TLR3 nuclear signal transduction initiated by TRIF binding to the TIR of the TLR3 homodimer. TLR3 monomers dimerize with binding of the dsRNA ligand. Activated TRIF initiates two pathways. The first results in the transitory induction of the IFNs. The second is a species variable pathway (rodents ≫primates) that operates though NFκB (dashed line), which transiently induces the production of inflammatory cytokines. The adapter protein cascade initiated by TRIF (TIR-domain-containing adapter-inducing interferon) includes TBK1 (TANK-binding kinase 1 binds to TRAF3), TRAF1/3 (TNF receptor associated factors), NAP1 (Nck-associated protein 1), IKK (IκB kinase), IKKε (inhibitor of IκB kinase), P13K (Phosphoinositide 3-kinase), IRF3/7 (interferon regulatory transcription factors), TAK1 (protein kinase of MLK family), TAB1 (TGF-β activated kinase 1), RIP1 (Receptor-interacting [TNFRSF] kinase 1), NFκB (nuclear factor kappa-light-chain-enhancer of activated B cells), IκB (inhibitor NFkB). The ectodomain of TLR3 consists of a horseshoe shaped structure populated by 23 leucine-rich β-sheets (orange disks) connected by non-ordered chains containing RNA binding residues. The transmembrane a-helices (solid orange) connect the ectodomain to the cytoplasmic TIR domain (dark green). The phosphorylated TIR binds TRIF to initiate the adapter protein cascade. B. Intracellular pathways for MyD88 dependent for TLR 1/2 and 1/6 heterodimers and TLR 4–10 homodimers with the diverse PAMP ligands represented by a green bar is not necessarily as accurate in placement as is dsRNA with TLR3 in 1A. TLR4 uses both the MyD88 dependent and independent pathways. Reproduced from Mitchell WM, et al .Discordant Biological and Toxicological Species Responses to TLR3 Activation. Am J Path 2014; 184: 1062–72.

Rintatolimod is a dsRNA that functions as an activating ligand for TLR3 (Figure 1(a)). Unlike other dsRNAs, the activity of rintatolimod is limited to TLR3 with no induction of the cytosolic helicases (Figure1(b)) [18,19]. The importance of this unique property of rintatolimod is a reduction of inflammatory cytokines that has limited the clinical utility of other TLR-activating ligands that use the inflammatory cytokine inducing MyD88-dependent pathway of intracellular signaling [20].

### Chemistry

**Figure 2.**
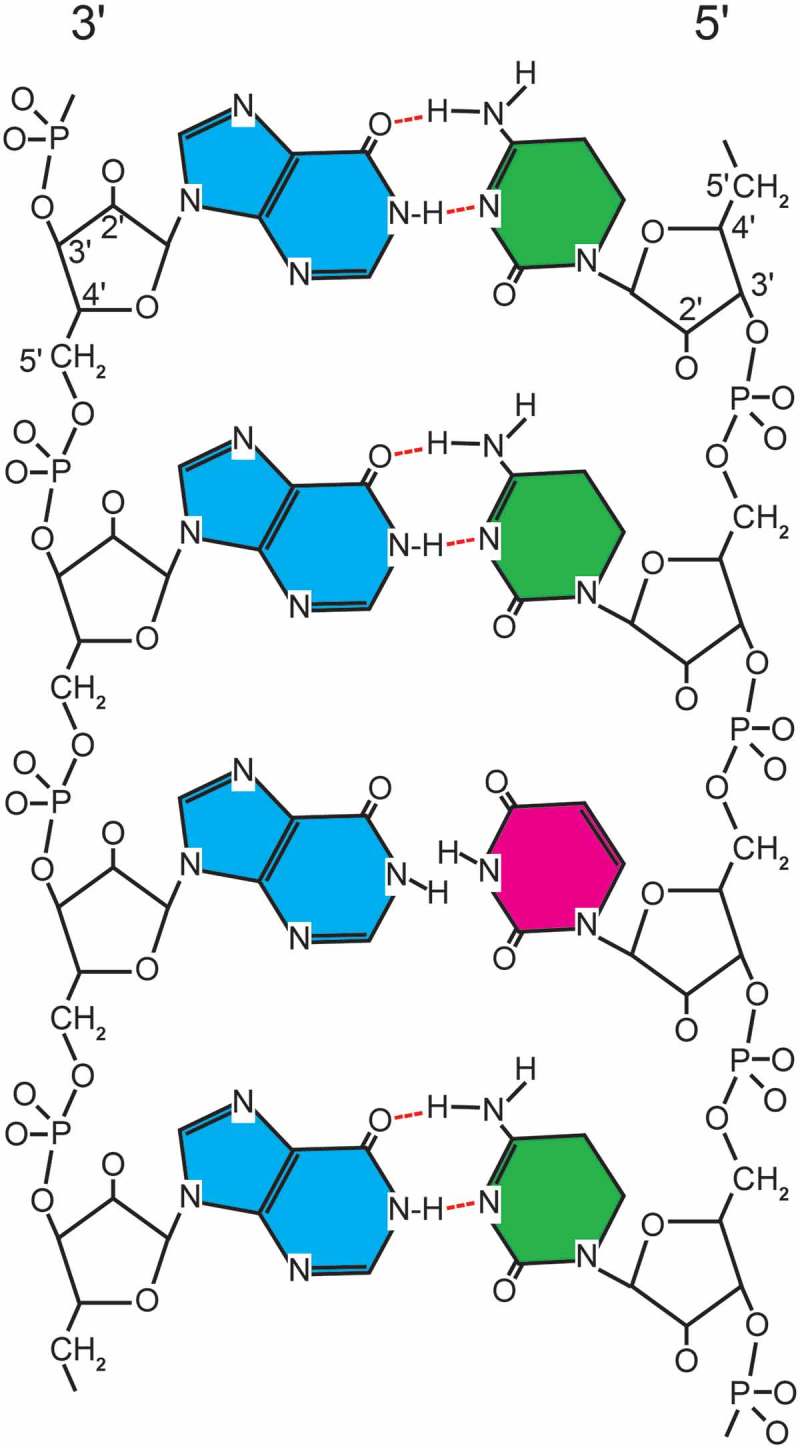
Diagramatic representation of rintatolimod. The dsRNA structure is maintained by hydrogen bonding. The introduction of a uracil into the poly C strand provides thermodynamic instability with an increased susceptibility to blood nuclease hydrolysis. The poly I strand is represented by blue (inosine). The poly C_12_U bases are represented by green (cytosine) and red (uracil).

Rintatoloimod is comprised of a single polypurine (inosine) strand hydrogen-bonded with a single polypyrimidine strand (cytosine) containing an inosine H-bond mismatched pyrimidine (uridine) for every 12 cytosines. These are assembled into a double-stranded RNA structure (Poly I: Poly C_12_U) that is maintained under physiological conditions by typical ‘Watson–Crick’ hydrogen bonding between purine and pyrimidine base pairs. The introduction of the pyrimidine base uridine at a 1:12 ratio (U:C) into the polypyrimidine strand maintains the overall double-stranded structure, but creates sites of thermodynamic instability (Figure 2) that allow rapid hydrolysis of Poly I: Poly C_12_U by serum nucleases to simple nucleosides compared to the parent Poly I: Poly C. Table 1 provides the specifications, for clinical grade rintatolimod, which includes the elemental composition, CAS registration number, nucleotide composition, chemical abstract names, other identifying names, and formulation. Rintatolimod for clinical use is provided as a sterile liquid formulation of high MW dsRNA polymer freely soluble under physiological conditions with a shelf life of over 7 years at 2–8°C.

**Table 1.** Characteristics and specifications for clinical grade rintatolimod.

**Generic name:** Rintatolimod
**Brand name:** Ampligen
**Molecular Formula**
Poly I: Poly C_12_U is designated by the molecular formula, [rI(13):rC(12)rU(1)]_n_ where: n = 46–138, or (C10 H11 N4 O7 P)_13_: (C9 H12 N3 O7 P)_12_ (C9 H11 N2 O8 P).
**Molecular Weight**
400,000–1,200,000 (10.0–15.0 S_20w_)
**Chemical Abstracts (CAS) Registry Number**
38640–92-5
**Chemical Name**
• Chemical abstracts names: 5ʹ-Inosinic acid, homopolymer, complex with 5ʹ-cytidylic acid polymer with 5ʹ-uridylic acid (1:1) (9CI) (CA INDEX NAME) 5ʹ-Cytidylic acid, polymer with 5ʹ-uridylic acid, complex with 5ʹ-inosinic acid homopolymer (1:1) (9CI) 5ʹ-Uridylic acid, polymer with 5ʹ-cytidylic acid, complex with 5ʹ-inosinic acid homopolymer (1:1) (9CI) Ampligen Poly(I).poly(C12U)
• Other names: Polyriboinosinic-polyribocytidylic 12: uridylic acid; Poly I: Poly C_12_U Rintatolimod
**Nucleotide composition**
The ratio of poly I to poly C_12_U is 0.9–1.1–1.0
**Formulation**
Rintatolimod is supplied in glass bottles as a biological active (TLR3 agonist in Ramos-Blue^TM^ reporter cells), sterile, colorless solution containing 200 mg of Poly I: Poly C_12_U in 80 ml of a physiological solution of salts (0.15 M NaCl, 0.01 M Phosphate, 0.001 Mg^++^) at a concentration of 2.5 mg/ml (2.25–2.70 mg/ml) with tertiary conformation demonstrated by circular dichroism. The product does not contain preservatives or antioxidants and is free of particulates (Particles ≥10 µm < 3000 per container; Particles ≥25 µm < 300 per container) and endotoxins (LAL activity < 1 EU/mL). The shelf life at 2–8°C is >7 years.

### Pharmacokinetics

**Figure 3.**
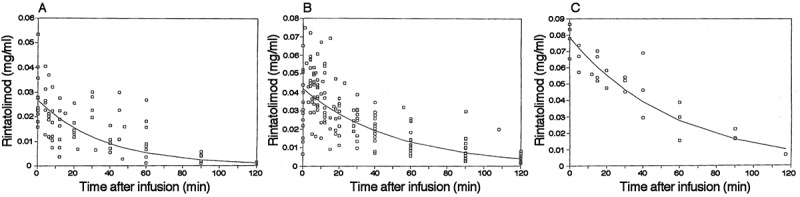
Representative rintatolimod blood elimination curves: (a) 200 mg (n = 6), (b) 400 mg (n = 12), and (c) 700 mg (n = 4) dose groups. Rintatolimod was delivered as a single bolus over an infusion period of 23–60 minutes. Rintatolimod and its metabolites (>50–100 nucleotides) were determined by hybridization employing a [^3^H]poly(C) probe. Each point is the average of duplicate concentration determinations at the designated dose and within 2.5 minutes of the time indicated. Each curve is the product of ‘n’ infusions, using first order decay kinetics. Revised from the doctoral thesis of Kenneth Strauss [25] with permission from the author.

The pharmacokinetic analysis of rintatolimod was conducted early in its clinical development under the auspices of a National Institutes of Health grant (CA29545) awarded to Hahnemann University. Extensive studies were conducted on the pharmacokinetics of rintatolimod administered as an intravenous infusion to human subjects Phase I/II clinical trials (CFS/ME and non-CFS/ME patients) [25,26]. Limited pharmacokinetic analysis was conducted in animals. Rintatolimod extracted from blood is in the dsRNA conformation and amenable to quantitation by a solution hybridization technique using a radioactive probe under chaotropic salt conditions which inhibit RNase degradation while allowing molecular probe hybridization displacement of the homologous RNA strand (>50 bp limit of detection). Since a minimum of 40–50 nucleotides are required for binding to TLR3 [27,28], the half-life measured in this assay is also the half-life for the ability of rintatolimod to induce innate immune responses. The assay is linear over a range of 3–530 μg/ml and has been validated in a multi-day study shown to be reproducible within 15% and to be 94% accurate [25,26]. There were a total of 132 patient-visits for which one or more of the derived pharmacokinetic parameters were available for analysis from the intravenous administration of 200–700 mg of rintatolimod over an average 30 minute infusion time (Figure 3). Immediate post-infusion analysis of rintatolimod and its metabolites showed an average of 60 ± 27 percent of theoretical maximum indicating significant degradation during the infusion period. First-order decay kinetics of rintatolimod from blood as a function of time is illustrated for infusion doses of 200 mg (Figure 3(a)), 400 mg (Figure 3(b)), and 700 mg (Figure 3(c)) and is consistent with an open one-compartment model. Since the poly(C_12_U)_n_ strand is more rapidly hydrolyzed than the poly(I)_n_ strand, the functional biological blood half-life (i.e. dsRNA 40–50 bp) is similar to the physical half-life measured by this probe hybridization method. The pharmacokinetic data show that drug accumulation arising from prolonged chronic treatment did not occur. Cmax and Cmax/Dose decreased with duration of treatment (non-CFS/ME) or remained constant (CFS/ME). Total exposure, AUC, was unaffected by duration of treatment for both disease groupings. Elimination half-life increased 60% with prolonged treatment time (>12 weeks); nevertheless, carryover is not observed since the longest half-life observed in any of the patients, 72 minutes, was still less than 1/50 of the shortest proposed dosing interval (72–96 hours, or twice/week). Gender as a factor in rintatolimod pharmacokinetics is unlikely to have any clinically significant impact on the selection of doses for the treatment of CFS/ME. Gender was detected as a statistically significant factor for clearance, Cmax and Cmax/Dose in selected populations. In none of the cases, however, were the differences (25–30%) likely to be clinically significant given the wide safety margin associated with the recommended dosing in CFS/ME patients. Age was not found to be a factor in the pharmacokinetics of rintatolimod. There was insufficient diversity in race in the pharmacokinetically evaluated population to permit the examination of the impact of race as a factor.

### Pharmacodynamics and mechanism of action

**Figure 4.**
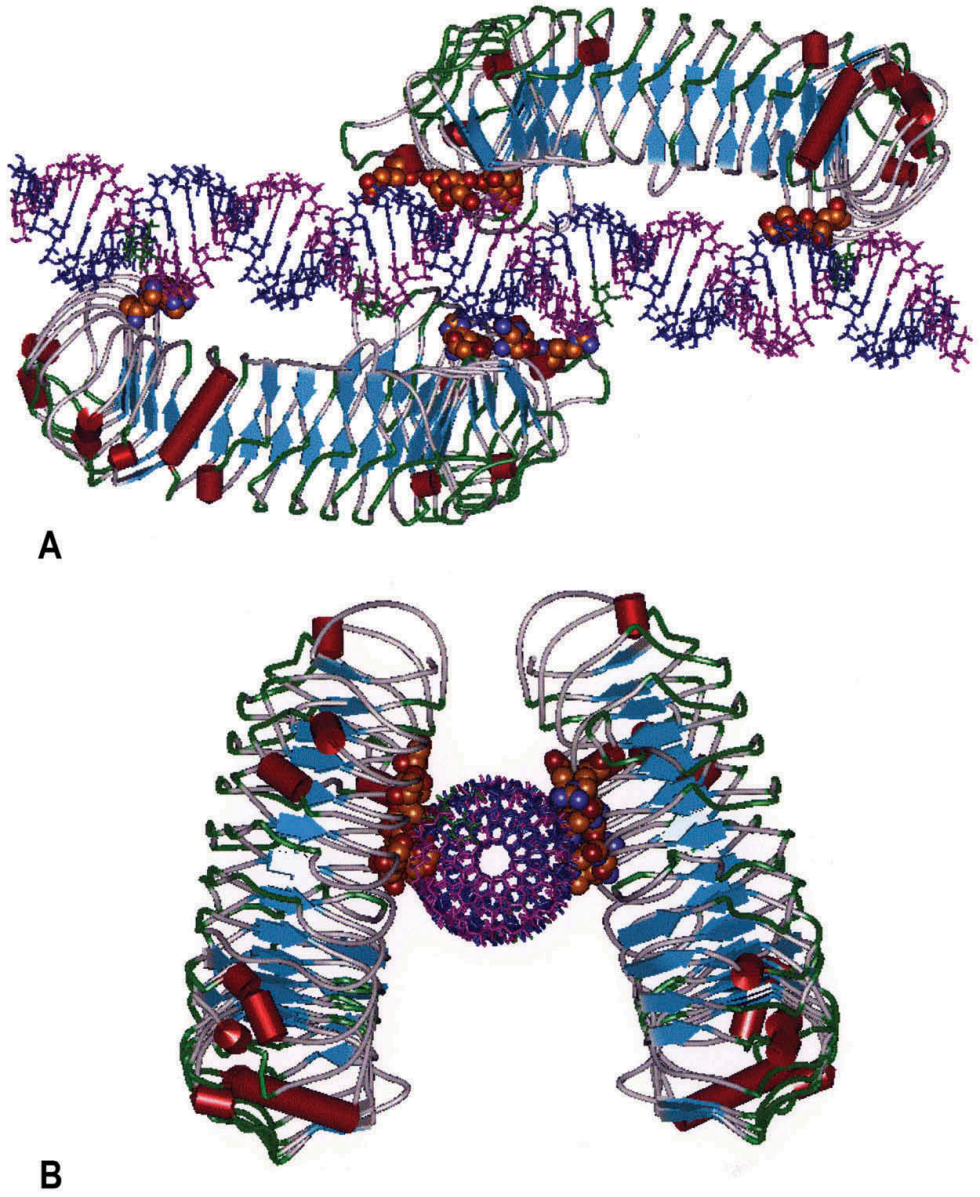
Molecular Model of the human TLR3 dimer ecodomain and its rintatolimod ligand. Figure 4(a) is viewed from a lateral view of rintatolimod bound to the active site of the TLR3 homodimer. The C-terminal regions of each dimer face each other and bind to the phosphate backbone of the dsRNA. The N-terminals of each TLR3 bind to opposite ends of the dsRNA with a minimum length of 45 bp required for interaction with essential residues of TLR3 for activation of intracellular signaling. Amino acids of TLR3 required for binding of rintatolimod are shown as CPK (Van der Waals’ radii) associated with the phosphate backbone. Figure 4(b) illustrates the TLR3 homodimer complexed with rintatolimod as seen down the long axis of the dsRNA. The TLR3 homodimers are represented as structural elements with the blue arrows signifying direction of β-sheets and the red cylinders signifying α-helices. The Poly I strand of rintatolimod is colored blue and the poly C12U strand magenta. Reproduced from Mitchell WM, et al. Discordant Biological and Toxicological Species Responses to TLR3 Activation. Am J Path 2014; 184: 1062–72.

TLR3 is activated by dsRNA [14]. The Toll-Like Receptors (TLRs) are a family of Class I transmembrane receptors (*n* = 10 in humans) that bind to pathogen-associated molecular patterns (PAMPs), which function as a first line of defense against microbial pathogens by the induction of innate immunity [29]. The PAMP for TLR3 is dsRNA detected in endosomes of antigen-presenting cells and on the cell surface of selected cells including endothelial cells and airway epithelium [30–32]. The expression pattern is consistent with sentinel activity for the detection of replicating virus in the host organism. Cellular location of TLR3 is modulated by UNC93B1 whose transcription is up regulated by dsRNA and which promotes trafficking of differentially glycosylated TLR3 to the plasma membrane [33]. Binding of dsRNA to TLR3 allows dimerization of TLR3 monomers and activation of a cytosolic phosphorylation that initiates a cascade of molecular events initiating transient activation of hundreds of genes [34]. Rintatolimod is restricted to TLR3 activation [18,19]. In contrast, other dsRNA configurations such as poly I:poly C activate the cytosolic helicases that utilize the pro-inflammatory MyD88 pathway. In contrast, TLR3 is the only TLR that exclusively uses a non-MyD88 pathway (TRIF) that minimizes the expression of systemic cytokines [20]. Figure 4 is a molecular model that illustrates rintatolimod-driven TLR3 dimer formation by non-covalent bonding. Studies with the dsRNA homopolymer, poly I:poly C, have demonstrated a positive allosteric affect dependent on the size of the dRNA [27] by lateral clustering [35]. Although TLR3 is activated with a minimum size of 40–50 bp [27,28], approximately 90 bp are required for the maturation of dendritic cells to mature antigen-presenting cells [36]. The release minimum MW specifications for rintatolimod take advantage of this allosteric property of TLR3 that favors activity with higher MW dsRNAs [27,33].

## Clinical efficacy

Of thirteen studies conducted with rintatolimod, nine were performed in severely debilitated CFS/ME patients (KPS ≤60). Three of these studies constitute the main studies of efficacy. Three were double-blind, randomized, placebo-controlled Phase II/III clinical trials at multi-sites (AMP-502, AMP-502 T, and AMP-516). AMP-502 T was a limited extension, which provided further efficacy and safety information. AMP-516 had a crossover in which subjects were initially randomized to active treatment or placebo followed by the blinded cross-over phase (AMP-516 C). All subjects received active treatment while remaining blinded to the initial treatment assignment. Five additional open label studies (AMP-501, AMP-504, AMP-509, AMP-511, and AMP-502E) measured safety and various efficacy parameters as well as long-term effects. Taken together, the entire database provides a consistent picture of durable activity in this population of severely disabled subjects and the accumulation of over 90,000 doses. More than 1200 patients have been enrolled in various rintatolimod studies in which over 830 unique CFS/ME patients received active drug.

In order to maintain direct comparability between rintatolimod clinical trials, only those subjects who met the inclusion criteria utilizing the original 1988 criteria of the CDC [37] and exceeding duration of the disease complex (1 year versus 6 months) were enrolled. Subjects enrolled in the Phase III clinical trial AMP-516 met both the CDC 1988 diagnostic criteria and the more relaxed 1994 CDC definition [38]. An international consortium proposed in 2011 that Myalgic Encephalomyelitis was a preferable term for the syndrome complex and in addition to the defining post-exertional exhaustion, diagnosis required additional evidence of neurological, immune, and energy impairments [39]. Although the Institute of Medicine has proposed recently Systemic Exertion Intolerance Disease (SEID) as a more descriptive name for CFS/ME [40], profound fatigue remains as the core descriptor for all definitions. The rintatolimod clinical trials focused on alleviation of that core symptom and its effect on quality of life.

The majority of patients had long disease durations of 6–9 years before enrollment that illustrates the chronicity of severe illness in some patients and for whom rintatolimod was targeted. Those patients randomized were representative of the severe disease state (KPS ≤60) and there were no important subsets of adult patients who were excluded. To date, no children have been studied.

### Clinical trials

A variety of test modalities have been used in assessing efficacy in open label and randomized, double-blind, placebo-controlled trials. They include Exercise Tolerance (ET) and Karnofsky Performance Status (KPS) as the primary modalities in evaluation of alleviation of the profound fatigue that affects activities of daily living. Other secondary test modalities included the SCL-90-R Short Check List-90-Revised-Cognitive Deficit Subscale, the Activities of Daily Living (ADL) index, the Short Form 36 (SF-36), Concomitant Medication usage, Investigator Assessment of CFS Signs and Symptoms, and Hospital/Emergency Room Admissions. Descriptions of these test modalities can be found in supplemental data.

#### Open label trials [41,42]

There have been five open-label studies of rintatolimod in CFS/ME, one of which is an on-going cost recovery study authorized by the FDA. These studies are summarized in Table 2. They provided guidance for the Phase II/III double-blind placebo-controlled trials. AMP-511 is a continuing open label treatment protocol that provides additional current safety data for rintatolimod in humans. AMP-516C was a cross-over trial at the conclusion of the placebo-controlled AMP-516 Phase III clinical trial [43]. Although technically an open label trial in which all patients received rintatolimod, the original arm of the parent AMP-516 remained blinded. There was no change in ET at 24 weeks (*p* = 0.58) for the patients remaining on rintatolimod. Patients switched from placebo to rintatolimod showed significant improvement in ET at 24 weeks (*p* = 0.04). Details of the open label trials are provided in supplemental data.

**Table 2.** Rintatolimod open-label studies.

Protocol	No. of patients treated	Percent female	Observed safety and apparent efficacy
AMP 501 Phase I	14	75	Drug was well tolerated throughout study (majority >24 weeks) in a relatively homogenous CFS/ME cohort. Efficacy observed in exercise performance, neurocognition, and anti-HHV-6 activity [41].
AMP 502E Phase I/ II	22	73	Open-label extension study in a 22 patient cohort who completed double-blind AMP 502 treated during an open-label extension phase for 1 year or longer. Drug was well tolerated throughout study (Phase 1 extention of administration time. Extention (Phase II) provided major improvements in Karnoksky Performance Status (*p* < 0.0001) [43].
AMP 509 Phase II	45	70	Belgian open-label study, with 44 patients evaluated. Similar dosing procedure and endpoints as AMP 502 [42]
AMP 511 Phase II/III	139	65	Ongoing cost-recovery, open-label study of Safety and Efficacy with similar dosing procedures and endpoints as AMP 502 [42]
AMP 516E Phase III	190	73	Partially blinded cross-over cohort extension of double-blind AMP 516 study [44]
Total	412	70	

#### Double blind, placebo-controlled, randomized multisite Phase II and Phase III trials

There have been three placebo-controlled clinical trials of rintatolimod in CFS/ME. AMP-502 was a Phase II study and AMP-516 was a Phase III study in which the primary endpoint achieved statistical significance (*p* ≤ 0.05). AMP-502 T was a small extension of AMP-502 under blinded conditions for dose escalation evaluation purposes. The trials are summarized in Table 3.

**Table 3.** Rintatolimod placebo-controlled studies [43,44].

Protocol	No. of patients	Percent female	Study design/data reported
AMP 502 Phase II	92	75	Placebo-controlled, randomized, multicenter Safety and Efficacy Evaluated Placebo (*n* = 47) or rintatolimod (*n *= 45): 200 mg for their first four doses, then 400 mg twice a week for 6 months
AMP 502 T Phase II	19	74	Placebo-controlled, randomized, multicenter Safety Evaluated 10 patients received placebo and 9 patients were treated with a higher dosing schedule of rintatolimod (400 mg three times a week)
AMP 516 Phase III	234	73	Placebo-controlled, randomized, multicenter, Safety and Efficacy Evaluated Placebo (*n* = 117) or rintatolimod (*n* = 117) 200 mg twice weekly for first four doses, then 400 mg twice weekly for 40 weeks (Stage I)
Total	345	74	

##### AMP-502

was a ninety-two patient, 24 week Phase II trial at four independent U.S. sites [24]. Study requirements were: age 18–60, KPS 20–60, restrictions on females of child-bearing age, and endurance levels verified by treadmill. The median KPS in both the placebo (*n* = 47) and rintatolimod (*n* = 45) treatment arms was 50 (‘requiring considerable assistance for daily care’) and the average duration of CFS/ME symptoms was 6.1 (rintatolimod) and 4.4 (placebo) years. General patient demographics for AMP-502 are summarized in supplemental Table S1. Patients assigned to each treatment group were well matched demographically and with respect to the severity of their illness with the exception of gender. There were no significant differences between treatment groups in the incidence of CDC CFS-defining symptoms, in the degree of patient debilitation as measured globally by means of KPS, or in their ability to perform routine activities of daily living measured by the Activities of Daily Living (ADL). The primary endpoint was KPS. Secondary endpoints were exercise tolerance (ET), ADL, SCL-90-R neurocognitive functional status, signs and symptoms, medications for CFS/ME, and hospitalizations or emergency room services.

A total of 84 of the 92 enrolled patients completed 24 weeks of treatment. Of the 8 dropouts, 3 of 4 placebo patients discontinued the study because of CFS/ME symptom intensification. The remaining 5 dropped out for non-medical reasons. At 24 weeks, patients receiving rintatolimod had statistically significantly greater improvements from baseline compared to the placebo cohort for global performance and perceived cognition (Table 4). The mean of the primary endpoint, KPS, was significantly improved (*p* < 0.001) as well as the median (*p* = 0.023). Statistically significant increases in the KPS score were observed in the rintatolimod-treated cohort compared to the placebo group at weeks 16, 20, and 24. At Week 24, the distribution of changes in KPS between placebo and rintatolimod cohorts showed 50% more responders in the rintatolimod cohort. Disease progression as measured by KPS was apparent in 6 of 47 (12.7%) in the placebo arm while 1 of 45 (2.2%) progressed in the treatment arm. Similar improvements were observed in the secondary endpoints associated with quality of life (cognition, ADL), exercise tolerance, and exercise work (O_2_ utilization). The objective quantitative improvement in the symptoms of CFS/ME observed with rintatolimod were seen also in the use of medications to alleviate CFS/ME symptoms. At the beginning of the study, patients were instructed to minimize all medications but were then allowed the use of prescription and over-the-counter drugs as needed. During the last 4 weeks of the study, the rintatolimod cohort used significantly fewer drugs to alleviate CFS/ME and CNS symptoms as well as pain as compared to the initial 4 weeks of study (Table 5). Forty-two percent (*n* = 39) of the patients had laboratory evidence of Herpes viral activation as demonstrated by giant cells expressing vital antigens on peri-pheral mononuclear cell (PBMC) culture. Sixty-nine percent were antigen positive for HHV-6, 8% cytomegalovirus, 5% herpes simplex, and 0% for Epstein-Barr virus. Expression of HHV-6 was associated with a poorer mean KPS score (50 v. 58, *p* < 0.02) although there were no differences in improvements observed in the rintatolimod cohort. Hospital emergency room admissions were dramatically improved in the rintatolimod cohort [42]. Fourteen of ninety-two enrolled patients were hospitalized or required Emergency Room services during the study. Significantly (*p* < 0.005), placebo patients were hospitalized or admitted to an emergency room for a total of 114 days compared to 7 rintatoloimod patients for a total 19 days (Table 6).

**Table 4.** AMP-502 clinical trial differential responses^a^.

	Percentage change (*n*)		
Parameter	Rintatolimod	Placebo	*p*-Value
KPS	+20 (41)	0 (43)	<0.001 (mean)/0.023 (median)^b^
Cognitive deficit	+27.3 (40)	+14.5 (43)	0.05^c^
ADL status	+23.1 (41)	+ 14.1 (43)	0.034^c^
Exercise duration	+10.3 (37)	+ 2.1 (39)	0.007^d^
Exercise work	+11.8 (37)	+5.8 (39)	0.011^e^

^a^Modified from [24] (Clinical Infectious Diseases/Oxford University Press).

^b^Median change (Week 24 vs. baseline) by Mann–Whitney test.

^c^An increased score quantifies a reduction in perceived deficit.

^d^ANCOVA with baseline as covariate.

^e^ANCOVA of log-transformed data with baseline as covariate.

**Table 5.** AMP-502 differential medication usages.

	Percentage change (*n*)		
Drug class	Rintatolimod	Placebo	*p*-Value^a^
CFS/ME symptoms	0.05	1.0	0.015
CNS symptoms	0.03	0.43	0.033
Pain	0.04	1.1	0.009
All medications	0.44	2.3	0.007

^a^Mean use during the first 4 weeks vs. last 4 weeks (*t*-test).

**Table 6.** AMP-502-relative incidence of hospitalizations^a^ in CFS/ME patients receiving rintatolimod vs. placebo.

		Rintatolimod	Placebo	Mann–Whitney
Number of admissions per hospitalized/ER patient	Mean	1.0	3.4	*p* < 0.005
	Median	1.0	3.0	
Number of days per hospitalized/ER patient	Mean	2.7	16.3	*p* < 0.005
	Median	1.0	18.0	

^a^Hospitalization was defined as either an emergency room admission or an admission to the inpatient service.

##### AMP-516

was a Phase III randomized, double-blind, placebo-controlled, multi-site (*n* = 12) clinical study [43,44]. There were a total of 234 well-matched (age, gender, CFS/ME duration, and body weight) patients equally divided between the two cohorts (*n* = 117) in a 40-week study in which 79% (*n* = 93) and 86% (*n* = 101) of the rintatolimod and placebo cohorts, respectively, completed the arduous clinical trial (40 weeks with IV infusions of ~35–40 minutes, twice per week). ET utilizing a modified Bruce treadmill protocol was the primary endpoint. At Week 40, there was a net improvement of 21.3% (*p* = 0.047) from baseline in the ITT rintatolimod cohort compared to the ITT placebo cohort (Table 7A). Mean ITT intrapatient cohort percent improvement of 36.5 % (*p* < 0.001) versus 15.2% placebo (*p* = 0.198) further supports a beneficial effect of rintatolimod on ET. Net ET improvement of 24.6% from baseline in the rintatolimod cohort was observed for the smaller (*n* = 93) 40-week rintatolimod trial completion group (*p* = 0.019) (Table 7B), and in those ITT patients without significant rintatolimod dose reductions net improvement was 28.0% at the end of the 40-week trial (*p* = 0.022) (Table 7C). In agreement with the ITT analysis, both the trial completion and those patients without significant lapses in drug administration demonstrated intrapatient improvement (*p* < 0.001) versus placebo (*p* > 0.24). Table 7D illustrates the effect of baseline ET stratification (≤9 minutes vs. >9 minutes) on ET performance at 40 weeks in the ITT population. Those patients able to achieve a > 9 minute duration on the CFS/ME-modified Bruce protocol at baseline and randomized to rintatolimod (*n* = 60) demonstrated a statistically significant advantage over placebo (*n* = 66) (*p* = 0.034) at 40 weeks although the lack of statistical efficacy in the ≤9 minutes cohort may be a lack of statistical power (*n* = 40 versus *n* = 60 between the placebo and rintatolimod sub-cohorts, respectively).

**Table 7.** AMP-516 analysis of the effect of rintatolimod on the primary endpoint, Exercise Tolerance (ET) [43,44].

A. Increase in exercise treadmill duration with rintatolimod in CFS patients (intent-to-treat)					
	Mean (SD) exercise duration (Seconds)		Percent increase from baseline^a^		
Study interval	Rintatolimod(*n* = 100)	Placebo (*n* = 108)	Rintatolimod (*n* = 100)	Placebo (*n* = 108)	*p*-value
Baseline	576 (257.5)	588 (234.4)	-	-	0.729^b^
Week 40	672 (314.1)	616 (286.7)	36.5	15.2	0.047^c^
*p*-value^d^			<0.001	0.198	
B. Increase in exercise treadmill duration with rintatolimod in CFS patients (trial completion population)					
	Mean (SD) exercise duration (Seconds)		Percent increase from Baseline^a^		*p*-value
Study interval	Rintatolimod (*n* = 93)	Placebo (*n* = 101)	Rintatolimod (*n* = 93)	Placebo (*n* = 101)	
Baseline	583 (254.7)	587 (237.3)	-	-	0.908^b^
Week 40	691 (311.4)	614 (291.2)	40.2	15.6	0.019^c^
*p*-value^d^			<0.001	0.244	
C. Increase in exercise treadmill duration with rintatolimod in CFS patients without significant dose reductions (intent-to-treat)					
	Mean (SD) exercise duration (Seconds)		Percent increase from baseline^a^		*p*-value
Study interval	Rintatolimod (*n* = 83)	Placebo (*n* = 98)	Rintatolimod (*n* = 83)	Placebo (*n* = 98)	
Baseline	581 (256.2)	590 (235.3)	-	-	0.813^b^
Week 40	690 (308.2)	616 (291.4)	43.0	15.0	0.022^c^
*p*-value^d^			<0.001	0.263	

D. Effect of baseline ET on Week 40 ET (intent-to-treat)								
	Mean (SD) exercise duration mean (seconds)				% Gain Rintatolimod over Placebo^a^		*p*-value^c^	
Baseline ET Strata (Minutes)	≤ 9 Drug (*n* = 40)	≤ 9 Placebo (*n* = 42)	> 9 Drug (*n* = 60)	> 9 Placebo (*n* = 66)	≤ 9	> 9	≤ 9	> 9
Baseline	321 (153.3)	353 (144.6)	747 (148.3)	738 (137.7)				
Week 40	450 (284.2)	446 (264.6)	820 (237.3)	725 (245.9)	31.0	15.0	0.517	0.034

E. Mean/Median baseline and Mean/Median change from baseline in exercise treadmill duration (seconds) at Week 24 (Stage 2) (ITT population)				
	Rintatolimod to Rintatolimod		Placebo to Rintatolimod	
Exercise tolerance parameter	Mean (SD)	Median	Mean (SD)	Median
Baseline ET (seconds)	706 (308)	726	626 (291)	638
ET at 24 weeks (seconds)	696 (323)	732	669 (288)	665
Percent improvement	22.9	-	39.0	-
*p*-value^c^	0.58	0.69	0.04	0.02

^a^Mean intra-patient percent improvement. ^b^Student’s *t*-test comparing mean baseline ET between treatment groups. ^c^Analysis of covariance (ANCOVA) with baseline as a covariate comparing the mean ET change from baseline within each treatment group. ^d^Paired *t*-test comparing whether the change from baseline is equal to zero within each treatment group.

**Figure 5.**
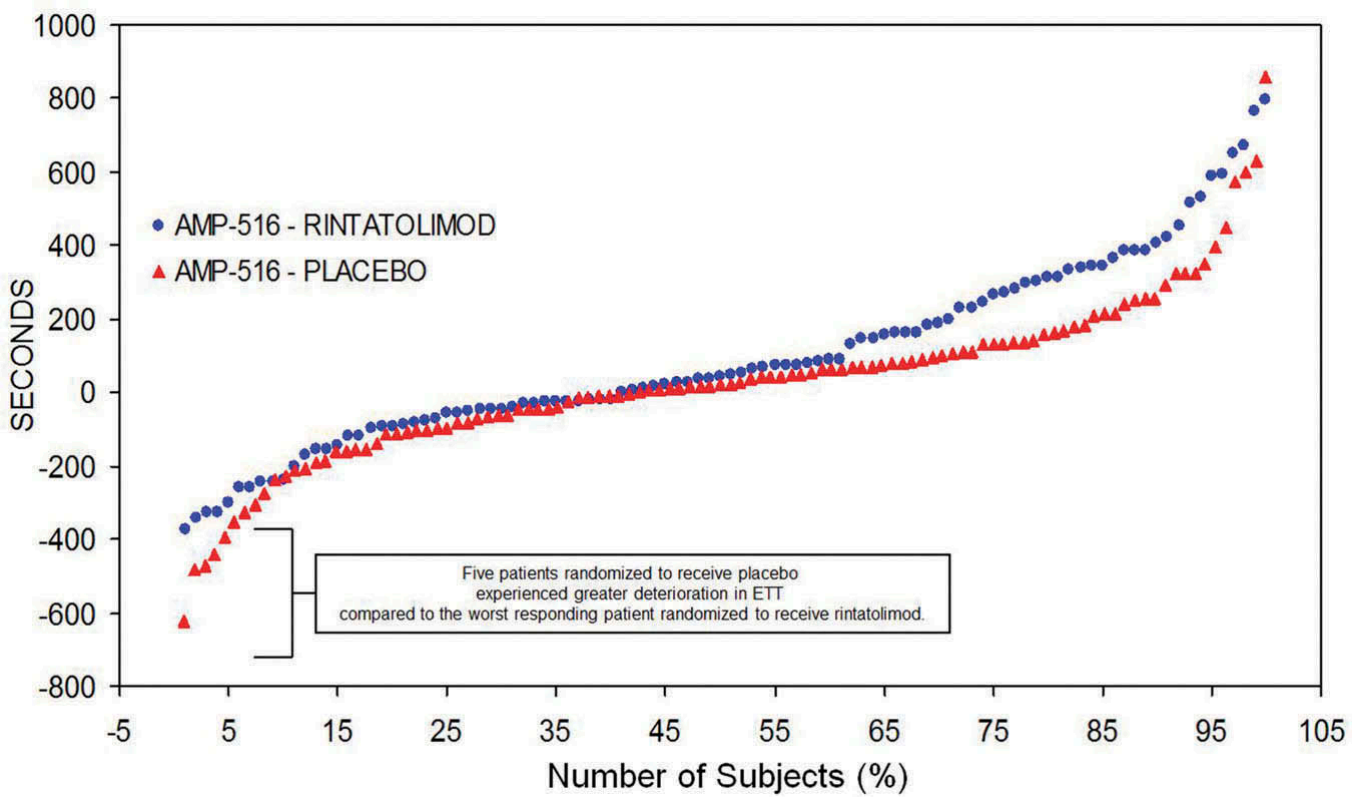
AMP-516 plot of ET difference from baseline in seconds at 40 weeks treatment (ordinate) per each patient (abscissa). Plot of ET difference from baseline in seconds at 40 weeks treatment (ordinate) per each patient (abscissa). Reproduced from Strayer et al. Chronic Fatigue Syndrome/ Myalgic Encephalomyelitis (CFS/ME): Characteristics of responders to Rintatolimod. J Drug Res Dev 2015;1: doi http://dx.doiorg/10.16966/jdrd.103.

The individual patient ET responses to rintatolimod compared to placebo for the ITT population is captured in Figure 5. Individual patient change in ET from baseline at 40 weeks is plotted from lowest to highest ET performance [44]. There is a minimum of three different ET response cohorts– a high response cohort, a minimal response cohort, and a negative response cohort. In the high response cohort (upper 40% on right side of the plot), there is a clear improvement in ET in the rintatolimod cohort versus placebo. The middle cohort represents minimal change between rintatolimod and placebo. The negative response cohort (on the left side of the plot) shows deterioration in ET performance in both rintatolimod and placebo patients. Nevertheless, rintatolimod appears to reduce deterioration in ET versus the placebo controls for the poorest modified treadmill responders at baseline.

Additional *post hoc* evidence supporting the efficacy of rintatolimod in CFS/ME was provided by an analysis of the frequency distribution of percent improvement in ET from baseline to Week 40 in the rintatolimod versus placebo cohorts (Table 8A). The proportions of patients in the ITT population with changes in ET from baseline to Week 40 of at least 25% and of at least 50% were 1.7 and 1.9-fold greater for patients randomized to rintatolimod than placebo, 39% versus 23% (*p* = 0.013) and 26% versus 14% (*p* < 0.028), respectively. CFS/ME patients further segregated into two ET cohorts. Dichotomization of ET ≥25% and ≥50% improvement from baseline within the subcohort, ET >9 minutes at baseline, further identifies responders to rintatolimod versus placebo (Table 8B) (*p* ≤ 0.004).

**Table 8.** Analysis of percentage of CFS/ME patients improving ET by at least 25% and 50% from baseline [43].

	% of Patients (*n*) improving		
Percent improvement	Rintatolimod	Placebo	*p*-value^a^
A. Intention to treat (ITT) population (*n* = 208)			
≥25%	39% (*n* = 39)	23.1% (*n* = 25)	0.013
≥50%	26% (*n* = 26)	13.9% (*n* = 15)	0.028
B. Subsets of ITT population with baseline ET>9 minutes (*n* = 126)			
≥25%	33.3% (*n* = 20)	12.1% (*n* = 8)	0.004
≥50%	23.3% (*n* = 14)	4.5% (*n* = 3)	0.003

^a^Probability values derived from the Chi-square test or Fisher’s Exact Test if any cell had less than 5 observations.

Impaired oxygen consumption, characteristic of chronically debilitated patients with severe cardiac dysfunction is also well documented in CFS/ME [45]. Decreased oxygen uptake is also an index of physical dysfunction in terms of reduced ambulatory skills, rapid onset of exertional dyspnea, and profoundly debilitating fatigue. The maximal oxygen utilization (VO_2_ max) in CFS/ME patients during exercise treadmill testing can also be used as a criterion for determining the seriousness of the disease and its evolution. The functional impairment of the AMP-516 CFS/ME subset population (~1/2 of drug and placebo cohorts) was determined by measuring maximal oxygen consumption during ET testing (Table 9) [39]. Despite the reduced statistical power for a subset analysis, rintatolimod improved VO_2_ max by 5.5% (*p* = 0.05).

**Table 9.** AMP-516 maximal oxygen utilization (VO_2_ max) in >9 minute baseline cohort.

	VO_2_ max mean (ml/min/kg)		Percent increase from baseline		
Week	Rintatolimod	Placebo	Rintatolimod	Placebo	*p*-value^a^
Baseline	22.39	21.70	__	__	__
	(*n* = 58)	(*n* = 60)			
Week 40	22.75	20.86	1.61	−3.87	0.05
	(*n* = 58)	(*n* = 62)			

^a^Analysis of Covariance (Baseline as Covariate), log transformed valves; High Stratum (Baseline > 9 minutes).

The original placebo cohort in a blinded cross-over Stage 2 of AMP-516 achieved a mean intra-patient percent improvement in ET of 39% (*p* = 0.04) at 24 weeks, while the original rintatolimod cohort maintained their improvement in ET (Table 7E).

Similar improvements were observed in the AMP-516 study secondary endpoints. Decrease in drug use was observed in both the ITT and study completion patients taking CFS/ME palliative drugs (*p* = 0.015 and *p* = 0.01, respectively). KPS, ADL, and SF-36 vitality scores were significantly improved from baseline (*p* < 0.01) in the rintatolimod cohort.

#### Correlation of clinical trial efficacy data

A total of 331 patients were evaluated and randomly assigned to either the placebo (*n* = 164) or rintatolimod (*n* **= 162) cohorts in two primary efficacy trials. Both the Phase II and Phase III double-blind, placebo-controlled trials achieved statistical significance of their primary endpoints, both physical performance based, KPS and ET, respectively. Both studies showed significant improvement in the primary symptom of CFS/ME, fatigue. Alleviation of profound fatigue was achieved as evidenced by significant improvement in ET or KPS in each controlled trial. Supportive evidence for improvement in the quality of life for the rintatolimod cohorts were reduced use of medications in an effort by patients to reduce the debilitating symptoms of CFS/ME and reduced hospital/emergency room admissions as well as SF-36 vitality and ADL score improvements. Perceived cognition was a secondary endpoint in AMP-502, which demonstrated statistical significant improvement versus placebo. Improvements observed in the placebo-controlled, double-blind clinical trials were observed in the open label trials, detailed in the supplemental data and provide further confidence that rintatolimod is clinically active in a substantial number of patients with CFS/ME.

## Safety and tolerability

### Human trial safety

Chronic administration of rintatolimod in clinical trials has been generally well tolerated. To date, over 90,000 doses have been infused intravenously. The major toxicities seen in a minority of patients during the first weeks of infusion are mild flu-like symptoms probably secondary to the induction of interferon. Table 10 provides all adverse events observed in the rintatolimod Phase II/III-controlled clinical trials in which there is >5% difference between drug and placebo cohorts. There were a total of 44 serious adverse events (SAE) observed, which were equally divided between the rintatolimod and placebo cohorts. In the opinion of the site principal investigators, none of the SAEs were definitely attributable to the study drugs (rintatolimod or placebo). One probable SAE occurred in the placebo cohort (Table 11). Summation of all SAEs (open label plus controlled clinical trials) demonstrates that 7.7% of patients receiving rintatolimod experienced a SAE compared to 8.6% of the placebo cohort [42].

**Table 10.** Summary of all patients with adverse events with at least 5% difference between rintatolimod and placebo.

Patients with any adverse events		Controlled portions of Phase II/III clinical trials	
		Studies AMP-502 and AMP-516	
		Rintatolimod	Placebo
		(*N* = 162)	(*N* = 164)
		161 (99.4 %)	160 (97.6 %)
Adverse events	% Difference*		
Flu-like symptoms	13.9	72 (44.4 %)	50 (30.5 %)
Headache	12.8	74 (45.7 %)	54 (32.9 %)
Chills	9.4	27 (16.7 %)	12 (7.3 %)
Fever	8.2	33 (20.4 %)	20 (12.2 %)
Vasodilatation	7.6	27 (16.7 %)	15 (9.1 %)
Pain	7.3	75 (46.3 %)	64 (39.0 %)
Injection site reaction	7.1	50 (30.9 %)	39 (23.8 %)
Pruritus	7.0	33 (20.4 %)	22 (13.4 %)
Diarrhea	6.3	36 (22.2 %)	26 (15.9 %)
Syncope	6.2	13 (8.0 %)	3 (1.8 %)
Ear disorder	5.7	22 (13.6 %)	13 (7.9 %)
Nausea	5.4	67 (41.4 %)	59 (36.0 %)
Migraine	−5.3	16 (9.9 %)	25 (15.2 %)

**Table 11.** Relationship of SAEs to study drug as determined by blinded investigators.

	Number of serious adverse events^a^	
Relationship to study drug	Rintatolimod	Placebo
Not related	16	16
Remote	4	3
Possible	2	2
Probable	0	1
Definite	0	0
Total	22	22

^a^Determined at time of occurrence.

### Comparative animal toxicities

Rintatolimod demonstrates significant reduced toxicity in primates versus other animal species [20]. Table 12 provides the relative species-dependent acute toxicity of rintatolimod. There is a two order of magnitude difference in the relative toxicities between rabbits and the cynomolgus monkey with dog and rat showing intermediate toxicities between the extremes. This relative toxicity differential is extended to sub-acute and chronic toxicity studies in rats versus primates. In the rat hematological, hepatic, bone marrow, and thyroid pathologies have been observed [20]. In the cynomolgus monkey, the only significant observed anomaly is in the thyroid and bone marrow. At higher doses (greater than equivalent clinical dosing), thyroid is associated with follicular hyperplasia and increases in plasma TSH and T4. Significantly no elevations of thyroid hormones have been observed in humans. Increased myelopoiesis has been observed in monkeys. No bone marrow examinations have been conducted in humans.

**Table 12.** Comparative species sensitivity to rintatolimod^a.^ [20].

Species	Maximum tolerated dose
Rabbit	1.25 mg/kg/dose
Dog	10 mg/kg/dose
Rat	12.5 mg/kg
Cynomolgus monkey	100 mg/k g

^a^The MTD is defined as the highest dose with no observed mortality or moribund toxicity.

There is no evidence that rintatolimod can be carcinogenic. RNAs are recognized as electrophilic targets for chemical carcinogens, but not as carcinogens themselves. Indeed, it is probably not possible for non-coding dsRNAs such as rintatolimod to be carcinogenic and this is supported by a comprehensive literature review. An extensive review of the current literature including standard toxicology/reference texts found no evidence that dsRNA can act as a carcinogen [42]. In addition, the National Toxicology Program lists no RNA as a suspected or proven carcinogen. Similarly, there is no evidence that rintatolimod can serve as a source for the generation of siRNAs or microRNAs [42]. Mutagenesis studies in four independent studies with rintatolimod were negative (Table 13) [42]. Classical two species, 2-year studies for carcinogenesis have not been done. However, a 6-month study in the rat and cynomolgus monkey provided no evidence of cancer or pre-cancer dysplasias in the multiple organs examined [42]. In rat, embryological studies indicated a potential for fetal death and miscarriage [42]. There has been no indication of teratological toxicity in rat [42]. In rabbits, a decrease was seen in fetal number and weight. Toxicity in female rabbits may be associated with malformations or defects in fetal rabbits [42]. The differential toxicities between rats and humans are correlated with relative systemic cytokine responses as well as differential blood half-lives [20]. Statistically significant dose-dependent cytokine differences between rats and non-human primates have been observed during the first week of rintatolimod administration as well as at Week 8 [20]. Human plasma has greater nuclease activity than other species and complements the estimates of relative species toxicities found in the comparative acute toxicity analysis (Table 12).

**Table 13.** Rintatolimod mutagenic potential.

Mutagenicity assay	Result
Mammalian Cytogenetic Assay (CHO Cells)	Negative
Ames Assay	Negative
L5178Y TK ± Mouse Lymphoma Mutagenesis	Negative
Mouse Bone Marrow Micronucleus Assay	Negative

## Regulatory affairs

The first CFS/ME patient treated with rintatolimod was at the request of the FDA. The dramatic response of this index patient resulted in an open-label clinical trial (AMP-501) [41] that yield results that mimicked the index patient. The success of that trial resulted in a major effort by Hemispherx Biopharma to bring the drug to market. During a multi-decade dialog with the FDA, rintatolimod has been sequestered with five separate review groups. Currently there is an open NDA with continuing dialog with the FDA. As detailed earlier, there have been two double-blind, placebo-controlled trials. Both trials achieved statistically significant improvements in their primary endpoints with minimal toxicities. The drug has not received a marketing approval despite the lack of proven efficacious agents in the treatment of this disease that can be severely debilitating and is estimated to affect over one million persons in the U.S. Rintatolimod has FDA orphan drug status with individual patient access to the drug with a treatment protocol for CFS/ME (AMP-511) in existence for nearly two decades. Recent *post hoc* studies have demonstrated that about 40% of CFS/ME patients can be expected to respond to rintatolimod while about 10% of the poorest responders appear to be retarded in deterioration of symptoms with time [44]. A CFS/ME-modified treadmill test provides an objective basis to select those patients most likely to have a vigorous response to rintatolimod.

## Discussion

Rintatolimod has been generally well tolerated and significantly improved physical performance primary endpoints in two double-blind, placebo-controlled clinical trials that are supported by five open-label trials, one of which was a cross-over of a Phase III trial that remained double-blinded as to original assignment group (active drug versus placebo). Rintatolimod is not active in all patients. Indeed, the multiplicity of patient primary and secondary responses to rintatolimod was not unexpected. CFS/ME is a syndrome with the common unifying symptom of profound fatigue. Approximately, 30–40% of severe CFS/ME patients can be expected to achieve some clinical benefit using the original CDC definition with severity defined as KPS ≤ 60. There is substantial evidence to support the hypothesis that individuals with persistence of debilitating symptoms are associated with a variety of inappropriate immune responses initiated by an intracellular pathogen and driven by dysfunctional gene responses that fail to clear or suppress the initiating agent. The differential responses observed with rintatolimod administration support this hypothesis. It is thus of interest to consider the potential of possible pathogens and modifying influences of dysfunctional gene expression and immune responses observed in CFS/ME.

### Acute infection and fatigue

The fatigue associated with acute infection is usually self-limited although some may have a prolonged course. Examples of the latter include Epstein-Barr virus (HHV-4), the causative agent of acute infectious mononucleosis, and *Borrelia burgdorferi* (post-treatment chronic Lyme disease). Recent proteomic analysis of cerebral spinal fluid (CSF) from persistence of these two infectious agents, however, distinguish patients with chronic Lyme disease from CFS/ME [46]. Studies linking contemporaneous laboratory diagnosis of acute infectious agents with the subsequent development of CFS/ME are rare although non-contemporaneous reports of a variety of infectious agents associated with CFS are common. An example of a prospective infectious diagnosis with the subsequent development of CFS/ME was established in a small community in Australia [47] using IgM to IgG seroconversion or rising IgG titers to Epstein-Barr virus (a DNA virus), Ross River virus (an alpha RNA virus), and *Coxiella burnetii* (an obligate intracellular bacterium and the causative agent of Q fever). Of the 253 patients with antibody evidence of acute infection with these 3 intracellular pathogens, 12% subsequently developed CSF/ME demonstrating that no single infectious agent is responsible for the syndrome and that induction of CFS/ME is dependent on factors other than primary infection. Similarly, in a small study CSF/ME patients with diagnostic high titer IgG titers to *Chlamydophila pneumoniae*, an obligate intracellular bacterium associated with atypical pneumonia, responded to anti-Chlamydial antibiotics with symptom resolution and declining antibody titers suggesting a cause and effect relationship [48]. There are a small number of case reports of the association of parvovirus B19 (a small ssDNA virus causing erythema infectiousum in children) and CFS/ME with resolution in three cases treated with pooled human immunoglobulin [49]. In a study involving 200 patients with CSF/ME, no differences between patients and controls were found in IgM or IgG titers against structural proteins of parvovirus B19 although 42% of patients versus 7% of controls had antibodies against the viral regulatory NS1 protein [50] suggesting a non-productive persistence of parvovirus B19. The affinity of polioviruses (enteroviruses) with their infection affinity for the CNS and GI tract has generated a number of studies linking CFS/ME with the enteroviruses. A large study of 165 CSF/ME patients exhibited the enteroviral VP1 structural antigen in 82% of gastric biopsies versus 20% of controls [51]. Mycoplasma have been demonstrated by PCR in PBMC in several studies [52,53] of patients with CFS/ME. Although effective antibiotic treatment is available, no antibiotic studies have been reported in CFS/ME associated with mycoplasma. Two studies linking retroviruses with CFS/ME have proven to be non-reproducible in the scientific community. The first described a retrovirus with sequence homologies to HTLV II [54]. More recently XMRV [55] and the closely associated polytrophic virus [56] have proven to be laboratory artifacts [57] although their reports generated premature attempts at therapy with anti-retroviral agents used for control of HIV. Similar to Epstein-Barr virus, most adults have antibodies to HHV-6 with no evidence of persistent-related disease. Unlike Epstein-Barr virus, however, numerous studies using culture and PCR have been reported linking HHV-6 with CFS/ME. In a study reported before a formal definition of CFS/ME had been developed, 70% of 259 patients with a CFS-like illness exhibited active HHV-6 replication in lymphocytes in primary culture [58]. The first patient treated with rintatolimod at the request of the FDA more than 2 decades ago had primary culture evidence of an active HHV-6 infection [59]. On the basis of that patient’s rapid response to rintatolimod, the first open label trial was initiated in CFS/ME patients with evidence of a HHV-6 infection that similarly showed significant activity [41]. The available HHV-6 data is consistent with the CFS/ME cohort responding to rintatolimod treatment. Conversely, those patients with evidence of Chlamydial infection responding to antibiotics may populate a TLR3 agonist (rintatolimod) non-responder cohort.

#### Multiple co-infections

Nicolson et al. [60] reported on the incidence of co-infections in 200 CFS patients and 100 controls by PCR analysis of whole blood. A total of 52% of CFS patients versus 6% of controls had evidence for the presence of at least one Mycoplasma species. A total of 7% were PCR positive for *C.pneumoniae* versus 1% in the controls and 30.5% positive for HHV-6 and 9% in the controls. Evidence for co-infection in Mycoplasma positive blood had similar incidences although there was no evidence of co-infections with these pathogens in the control cohort. One might logically expect a mixed non-cleared infection to respond to rintatolimod variably similar to the current mid-third minimal responders.

Despite two decades of attempts to identify specific infectious agents as the initiator of the signs and symptoms of CFS/ME, it is apparent that a multiplicity of obligate intracellular pathogens are capable of disease initiation. Although the pathogenesis of persistence in a minority of affected individuals has remained unclear, dysfunctional genetic responses involving the immune system and energy metabolism have been linked to the CFS/ME phenotype.

### Immune markers

Consistent with the evidence of infection with multiple obligate intracellular pathogens are the increased numbers of activated cytotoxic CD8+ cells observed in CFS/ME [61]. Although NK cell numbers are generally in a normal range, NK cell function is decreased [62]. The IFN inducible 2ʹ-5ʹ adenylate synthetase/ RNase L pathway is dysfunctional in CFS/ME. Constituitive RNase L is activated by 2ʹ-5ʹ oligomers of adenine that results in cellular mRNA degradation. 2ʹ-5ʹ adenylate synthetase (2ʹ-5ʹA) transiently induced by Type 1 IFNs requires dsRNA for activation. In CFS/ME there is as much as a log increase in bioactive 2ʹ-5ʹA with a concomitant reduction in latent 2ʹ-5ʹA and increase in the bioactivity of RNase L in PBMCs [63,64]. A clinical evaluation demonstrated that 46 of 73 patients with CFS/ME had an elevation of RNase L activity that was associated with a significant (*p* < 0.001) decrease in ET [65]. The dramatic increase in bioactive 2ʹ-5ʹA has been substantially linked to a 37kD proteolytic fragment of the expressed 83kD enzyme [66,67]. The up-regulation of 2ʹ-5ʹA synthetase/RNase L pathway is consistent with an antiviral response pathway that has been rendered dysfunctional by aberrant PBMC intracellular proteolysis. The latter is the result of 2ʹ-5ʹA synthetase dysfunction in some patients with CFS/ME with the production of dimers rather than higher oligomers of 2ʹ-5ʹA that inhibit proteolysis of the native RNase L [68]. Although the existing methodology is research lab-based and not suitable for high volume reference or hospital-based laboratories, the relative abundance of the 37kD RNase L fragment relative to the intact 83kD enzyme has been suggested as a clinical lab assay for CFS/ME [69,70]. The observed normalization of this dysfunctional immune response to viral activation by IFN further suggests correction by rintatolimod of a viral initiation phenomena in CFS/ME.

### Genetic markers

Differential gene expression in peripheral blood of CFS/ME patients has been reported by a number of investigators using DNA microchip analysis [71–81]. Recently, rigorous patient selection and a microchip that surveys the entire human genome coupled with qPCR gene validation has provided a more complete appreciation of the gene expression profiles that occur in CSF [9,82]. A complex array of differential gene expression can be categorized into functional subsets relating to responses to infection, immunity, inflammation, apoptosis, neurological function, and cancer [79]. Many of these differential gene responses are consistent with the large number of infectious agents linked with CFS/ME as well as the altered immune responses and variety of signs and symptoms observed with the disease. For example, eIF4G1 is an eukaryotic mitochondrial translation factor utilized in replication by a variety of viruses including the enteroviruses implicated in the pathogenesis of CFS/ME [83]. EIF4G1 variant 5 (GenBank:NM_004953) is up-regulated in CFS/ME suggesting a physiological response to viral replication as well as a gene variant favoring pathogen persistence. Similarly, genes associated with Epstein Barr Virus infection have been demonstrated recently to be up-regulated in most Kerr CSF/ME subtypes [84]. Patients with fatigue associated with Q-fever have similar gene expression profiles with CSF/ME [84].

Differential gene responses have been analyzed as a function of exercise tolerance in CFS/ME. Whistler et al. [73,74] associated exercise-dependent gene expression with the specific Gene Ontology categories of chromatin and nucleosome assemblies, cytoplasmic vesicles, membrane transport, and G-protein-coupled receptor. Distinct differences as a function of exercise have been demonstrated between CFS/ME, fibromyalgia, and multiple sclerosis [85,86].

### Mitochondrial function markers

Abnormal mitochondrial function [87] and structure by light and electron microscopy [88] have been observed in CFS/ME and consistent with reported muscle oxidative damage [89] and acetylcarnitine [90] and carnitine serum deficiencies [91]. A novel deletion in mitochondrial genes associated with energy production has been reported in a CFS/ME patient distinct from a common 4977 bp deletion observed in overt mitochondrial diseases [92]. The common 4977 bp deletion in mitochondrial DNA in CFS/ME has been reported 150–3000 times normal controls [93]. Rapid muscle intracellular acidosis in a CFS/ME patient detected by 31P nuclear magnetic resonance (31P-NMR) spectroscopy was suggestive of impaired oxidative metabolism [94]. Subsequent 31P-NMR studies demonstrated reduced biosynthesis of phosphocreatine [95] and lower levels of intracellular ATP [96] post-exercise. An ATP profile test consisting of ATP cytosolic transfer, oxidative phosphorylation efficiency, and the efficiency of ADP concentration within mitochondria from the cytoplasm in neutrophils has been reported [87]. Delivery of mitochondrial ATP to the cytoplasm as a function of CFS/ME severity as compared to normal controls demonstrated a clear discrimination of CFS/ME severity (*p* < 0.001) with no overlap with controls. The ATP assay suggests that the mitochondrial dysfunction in CFS/ME is multi-factorial and provides a rational basis for the exercise intolerance observed in CFS/ME and the differential response in exercise tolerance to rintatolimod [44].

### Rintatolimod and CFS/ME markers of disease

Rintatolimod is clearly active in improvement of ET and quality of life in a subset of patients with CFS/ME. Moreover, the clinical evidence suggests that rintatolimod inhibits disease deterioration in a minority of patients who fail to show improvement from activation of TLR3. The basis for this differential response to rintatolimod is unknown but may be secondary to a variety of microbes coupled with a combination of genetic polymorphisms resulting in an immune response unable to clear either a productive microbial infection or an activated non-replicative intracellular microbe. The diagnosis of CFS/ME remains a diagnosis of exclusion. To date, there have been no laboratory-based markers for CFS/ME diagnosis although there have been potential candidates that unfortunately have not been adapted by clinical reference laboratories. Prime examples include a dysfunctional 2ʹ-5ʹ adenylate synthetase (2ʹ-5ʹA) and functionally inactive NK cells reported in CFS/ME by multiple investigators. RNAse L has been observed by De Meirlier to be corrected by rintatolimod in patients (AMP-509 trial) [42].

Functional impairment of NK cells has been studied by multiple investigators and found to be up-regulated by rintatolimod *in vitro* [62] although the necessary validation in patient trials has not been accomplished to date. Moreover, the functional research assay used (Cr^51^ release from NK target cells) needs to be coordinated with the large number of NK cell markers that are available for flow cytometry on fixed cells. Despite the obvious need for inexpensive laboratory markers to aid clinicians in the diagnosis of CFS/ME as well as indicators of rintatolimod efficacy, there has been no national priority established for development.

Response to rintatolimod is apparently related to a multifactorial pathogenic basis with a variety of reported intracellular pathogens, hormonal, and immunological abnormalities linked with a variety of genetic signatures. Those patients able to reach 9 minutes on a CFS/ME-modified Bruce protocol are evidence-based responders to the drug. About 10% of CFS/ME patients with the poorest exercise tolerance may experience less disease progression. No drug is approved for the CFS/ME indication and I know of no other pharmaceutical company with drugs in advanced development despite FDA attempts to promote development by the pharmaceutical industry.

## Expert commentary

TLR3 is unique in its induction of innate immune responses. The exclusive use of a non-MyD88 pathway limits expression of inflammatory cytokines especially in humans as compared to non-primate animal models. Rintatolimod further extends this unique property by its lack of stimulation of the cytosolic helicases utilizing a non-TRIF signaling pathway observed with non-mismatched dsRNA polymers. Although significant improvements in physical performance primary endpoints has been seen in the Phase II/III trials and is supported by open label trials, the final regulatory approval may require objective laboratory diagnostics that identify patients most likely to respond to rintatolimod. Fatigue is the universal symptom of this disabling condition and is a common symptom in a multiplicity of human diseases (examples include multiple sclerosis, cancer, severe anemia, hypothyroidism). Table 14 lists the most likely diagnostic markers to identify responders to rintatolimod and their current developmental status. Rintatolimod has an extensive safety record in humans in which primates demonstrate significantly fewer toxic manifestations of a restricted TLR3 activation than non-primates. CFS/ME is a complex syndrome with a variety of diverse factors including gene expression related to disease induction. The identification of markers that can be adapted to clinical reference laboratories to identify rintatolimod responders would provide objective criteria for non-CFS/ME physicians and should provide a regulatory pathway to approval in CFS/ME.

**Table 14.** Evidence-based potential diagnostic markers for rintatolimod response in CFS/ME.

Marker	Rationale	Improvements
ET>9 minutes on modified Bruce protocol	Demonstrated in Phase II and Phase III clinical trials	ET under measurement of O_2_ utilization and CO_2_ production to insure acquisition of ‘oxygen debt’ as objective evidence of termination due to exhaustion
Low NK cell function enhancement	Low NK cell functional activity demonstrated in multiple studies. Low NK cell activity up-regulated with rintatolimod *in vitro*	*In vitro* response has not been correlated with *in vivo* response in CFS/ME patients. Cr^51^ release assay from NK cell target cells *in vitro* is a research lab assay. NK cell markers used in flow cytometry with fixed cells needs correlation with Cr^51^ release response to rintatolimod
37kD RNAse L	Low MW proteolytic form of RNAse L in CFS/ME demonstrated in multiple studies. Response to rintatolimod demonstrated in patients. Alternatively, dimer/trimer (and above) ratios can be determined and quantified	Research methods of analysis are not suitable for clinical reference laboratories. Identification of 37kD/83kD forms should be amenable to mass spectrometry analysis as well as 2ʹ-5ʹA oligomers
Mitochondrial dysfunction	Assayed in PBMCs from a single laboratory in multiple reports. Observed in muscle by the same laboratory	Assay needs independent validation. Early ET oxygen debt experienced by CFS/ME patients supports data in PBMCs
Multiplex PCR analysis of mRNA and SNPs in literature identified genes associated with CFS/ME	Platforms supporting multiplex analysis for dozens of identified genes are available that allow simultaneous quantitative analysis of genes implicated in CFS/ME	Key genes can be identified with practical analysis using multiplex platforms available in clinical reference laboratories
Massive parallel sequencing	Identification of microorganisms in plasma and/or PBMCs by Next Generation Sequencing (NGS). Human sequences easily distinguished from non-human	New NGS platforms are currently reaching clinical reference laboratories with significant reduction in costs. Potential for identification of new pathogens responsive to rintatolimod. New SNPs identifiable

## Five-year view

Rintatolimod has been demonstrated to reach statistical significance in two randomized, double-blind placebo-controlled clinical trials with patients with well-defined disease. No other pharmaceutical is in apparent development for this woefully neglected disease. Orphan drug status and the recent award of a new form and substance patent [97] for rintatolimod provides extended commercial viability. Rintatolimod, a restricted TLR3 agonist, is clearly active in a subset of CFS/ME patients and appears to reduce further disease deterioration in those patients who fail to improve physically. Genetic and immune markers identified (Table 14) may provide insights to other drugs for combinatorial CFS/ME therapy in which rintatolimod alone is non-efficacious. The use of Next Generation Sequencing of plasma and PBMN cells is needed to identify unknown systemic infectious agents such as from the gut microbiome that may be part of the etiological process in some patients with CFS/ME.

## Key issues

- Rintatolimod is a high MW synthetic mismatched double-stranded (ds) RNA (Poly I: Poly C_12_U) polymer
- Rintatolimod activates TLR3 that induces innate immune responses and initiates adaptive immunity
- All Toll-Like Receptors with the exception of TLR3 activate the pro-inflammatory cytosolic MyD88 pathway
- TLR3 uniquely activates the cytosolic TRIF pathway with limited pro-inflammatory responses especially in primates
- Rintatolimod is a restricted TLR3 agonist with no activation of the cytosolic helicases that use a non-TRIF pathway
- Restriction of rintatolimod provides an improved safety profile compared to other dsRNA agonists
- Rintatolimod has achieved statistical significance in randomized Phase II and Phase III double-blind, placebo-controlled, multi-site clinical trials in which the drug was administered IV bi-weekly for up to forty weeks in patients with severe CFS/ME
- Rintatolimod was generally well tolerated with adverse events randomly distributed between drug and placebo cohorts with the exception of initial mild flu-like symptoms in some patients
- Approximately 30–40% of CFS/ME patients can be expected to respond beneficially to rintatolimod

## Supplementary Material

SUPPLEMENTARY_DATA.pdf

## Supplemental data

The supplemental data for this article can be accessed here.
