# Supplementary material for: Efficacy of rintatolimod in the treatment of chronic fatigue syndrome/myalgic encephalomyelitis (CFS/ME)
Source: Expert Rev Clin Pharmacol. 2016 May 25;9(6):755–70. doi: 10.1586/17512433.2016.1172960 (PMC4917909; doi:10.1586/17512433.2016.1172960)
Supplement: SUPPLEMENTARY_DATA.pdf [file ierj_a_1172960_sm0041.pdf]

## SUPPLEMENTARY DATA

A. Open label clinical trials:

1) AMP-501 was the initial clinical study of rintatolimod in CFS/ME of safety and efficacy [S1]. Fifteen of sixteen screened patients (mean age 44 years and of which 73% were female) who fit the criteria of the Center for Disease Control (CDC) definition of CFS and had evidence of severe reduction in performance levels by low KPS of 20-60 (mean=47) and unable to work, were treated for a minimum of 16 weeks (25 months average) with intravenous (IV) infusions of rintatolimod. At baseline, 13 of the 15 treated patients showed evidence of cerebral dysfunction by lower than normal scores on the Wechsler Memory Scale (WMS) and lower than expected full-scale Wechsler Adult Intelligence Scale-Revised (WAIS-R) intelligence quotient (IQ) scores. The majority of patients (14 of 15) had anatomical brain abnormalities as detected by Magnetic Resonance Imaging (MRI). Human herpes virus-6 (HHV-6) was identified in giant cell co-culture assays from 100% of 14 patients who were evaluated at baseline. All evaluable patients displayed reduced performance during exercise tolerance (ET) testing, including oxygen consumption. These patients represented a relatively homogenous subset of CFS/ME patients with especially severe and sustained symptomatology that reflected the original index patient. All patients initially received 200 mg doses of rintatolimod, administered intravenously twice a week for variable periods of time. With no evidence of toxicity the dose was escalated to 400 mg twice weekly. Individual patients were subsequently increased to 300 mg twice weekly to 400 mg three times a week. 86% of the patients were treated for more than 24 weeks (one patient for more than 93 weeks). Thirteen of the fifteen patients showed objective evidence of beneficial response to rintatolimod, which was well tolerated without evidence of any cumulative toxicities that would require either a dose reduction or discontinuation.

Efficacy was evaluated by ET, neurocognative function, positive symptom distress index, and quantitative HHV-6 analysis (virus induced giant cells in peripheral blood and monoclonal antibody (mAb) detection of HHV-6 expressing cells). Duration and oxygen consumption was improved with rintatolimod treatment ($p=0.006$ and $p<0.0001$, respectively). The Wechsler memory scale was improved ($p=0.003$). The Positive Symptom Index indicative of psychological dysfunction as a function of rintatolimod treatment was not affected ($p=0.44$). In contrast, the CFS/ME symptom specific dimension was decreased significantly ($p<0.0001$). HHV-6 giant cells were significantly reduced ($p<0.0001$) as well as mAb identified HHV-6 infected cells ($p<0.01$).

2) AMP-502E was an open-label extension of the randomized, placebo-controlled, clinical trial of AMP-502 [S2]. Twenty-two CFS/ME patients entered an extension, open-label phase and received a minimum of one year and a mean of two years of additional rintatolimod treatment at the same dosage level utilized in the placebo-controlled AMP 502 study (400 mg twice weekly). Twenty-two CFS subjects were enrolled and Karnofsky Performance Status (KPS) was determined every four (4) weeks. Of the twenty-two (22)

subjects enrolled, one cohort of fifteen (15) patients (Group A) was selected based on the degree of responsiveness at the end of 24 weeks of treatment in AMP 502. To be included in this cohort, each patient had to show a ≥ 20 point KPS improvement at Week 24 compared to Baseline, or a more moderate (≥ 10) increase in the KPS score at Week 24 compared to Baseline that was accompanied by an improvement in the cognitive deficit subscale of the SCL-90-R of 0.8 or greater. The other cohort of seven (7) CFS/ME patients (Group B) independently chose to enter the extension (AMP 502E) phase of AMP 502 and no specific improvement in KPS scores was required for Group B. Group A had a mean duration of treatment of 115 weeks and a KPS at Baseline of 47.3 indicating that these CFS/ME patients required considerable assistance and in some cases were disabled, unable to care for themselves and required special care and assistance prior to entering the AMP-502 study. After the first 24 weeks of randomized, placebo-controlled treatment the mean KPS had increased 16.5 points (p<0.0001) and by the end of treatment in AMP-502E, KPS had increased a total of 31.3 points (p<0.0001).

The improvements for Group B were even greater during the extension phase. Baseline KPS was 53.33 indicating that the average Group B participant required considerable to occasional assistance for completing normal everyday activities of daily living. After a mean of 78 weeks in AMP- 502E, KPS increased 20 KPS points from the end of the randomized, placebo-controlled phase to the end of AMP-502E. The total mean improvement in KPS was +26.67 KPS points (p<0.0001).

The mean Baseline KPS for the entire group (n=22) prior to any treatment was 50, (requires considerable assistance for daily care). The mean KPS after the extension treatment was 80 (normal activity with effort; some signs or symptoms of disease). Time-in-study had a progressively palliative effect. Spearman ranked-order correlation (R=0.91, p=0.0001) indicating a strong inverse relationship between the baseline KPS and the percent of KPS improvement over Baseline at the end of the extension phase.

3) AMP-509 was an open label, single center (Belgium) study of rintatolimod (200 mg initial then 400 mg) administered by IV route twice weekly in 44 CFS patients (including 3 dropouts) [S3]. KPS and ET (bicycle) were used to evaluate physical performance and to assess quality of life and general health functioning. Other endpoints included neurocognitive functional status determination with the SCL 90-R and the SF-36 questionnaire, CFS signs and symptoms, and the ADL. Other parameters included an immune panel and safety assessment.

Table 3 summarizes the observed effect of rintatolimod in severe CFS/ME. The mean KPS score in the intention-to-treat CFS/ME cohort had dropped to near 50 ("requiring considerable assistance for daily care") and the average duration of CFS symptoms was 8.5 years). KPS mean baseline of 54 by week 24 had improved >17 points (p<0.0001). Similar improvements were observed in daily living functions (ADL), vigor (SF-36), and exercise performance (ET). The classical signs and symptoms were significantly improved as were assessments of cognition (SCL-90-R). The effects were durable on re-assessment at 3 years post-study.

4) AMP-511 is an on-going, prospective, multi-site study for continual evaluation of safety of rintatolimod in CFS/ME [S3]. To date over 130 patients with severely debilitating CFS/ME have received rintatolimod, which has been well tolerated with no serious

adverse events including patients with over 2 years of rintatolimod infusion (400 mg twice weekly). Eighteen (18) subjects had completed the first 24 weeks of the AMP-502 study. The KPS of these 18 patients in AMP 511 increased 22% from a mean KPS at Baseline of 50.6 to a KPS at Week 24 of 61.7. An intent-to-treat analysis of all 20 patients (includes carried forward KPS data on two patients (007 PRR and 010 SLD) who dropped out of the study prior to completing Week 24 shows the same result. Mean KPS increased 22% from 51 at Baseline to 62 at Week 24. The improvements in KPS correspond to a patient requiring considerable (daily) assistance for completion of normal activities of daily living at Baseline and following 24 weeks of rintatolimod, requiring only occasional (once or twice a week) assistance. Analysis of an activity monitor (Actigraph Model 7164) in this cohort demonstrated close correlation between the two methods of analysis ((Spearman's Correlation Coefficient = 0.97; p=0.0012).

5) AMP-516E was an original cohort double-blinded cross-over (Stage 2) to rintatolimod following the completion of the randomized Phase 3 (Stage 1) double-blinded, placebo controlled CFS/ME clinical study [S4]. 81% (n=73) of the rintatolimod Stage 1 completion cohort elected to continue on active drug compared to 90% (n=90) of the Stage 1 placebo completion cohort for 24 additional weeks. The placebo to rintatolimod Stage 2 cohort demonstrated a dramatic 39% improvement in ET (p=0.04) at 24 weeks while the rintatolimod to rintatolimod cohort maintained their ET status (p=0.58) indicative of a maximum drug response.

B. CFS/ME Instruments In Evaluation of Efficacy

1) Karnofsky Performance Status (KPS) is a global physician evaluation of the patient's ability to conduct activities of daily living and is sensitive to effective therapeutic intervention in chronic disease states, especially where patient functional status declines in association with severe debilitation [S5-S7]. Supplementary Table S2 illustrates the basis of scoring. Each 10 point change in KPS status represents a major change in medical/physical patient status.

2) Exercise Tolerance (ET) is an objective measurement of endurance using either a treadmill or bicycle with reduced performance standards to accommodate the reduced ability for CFS/ME patients to safely perform a measurable time with increased precision (Supplementary Table S3). Patients with CFS/ME frequently experience rapid onset of "oxygen debt" where anaerobic glycolysis exceeds the oxygen utilizing Kreb's cycle resulting in inefficient ATP production [S8]. In some studies oxygen consumption was performed and correlated with KPS.

3) SCL-90-R Short Check List-90-Revised-Cognitive Deficit Subscale is a self-assessment of cognition. Eight specific questions, which comprise the cognitive test of the SCL-90-R (Supplementary Table S4) are embedded within a series of 90 other items. Patients reported the level of impairment or distress produced by each of the eight items on an ascending unitary scale of 0, 1, 2, 3, 4, where "0" indicated the absence of impairment, "not at all"; 1, "a little bit"; 2, "moderately"; 3, "quite a bit"; and "4" reflected extreme impairment. The cognitive test score is the average of the patient's response to all eight items. Thus, a lower test score indicated less cognitive impairment than a higher

score. Although testing of cognition can falsely elevate estimates of cognitive ability based on “practice”, embedment in the SCL-90-R minimizes practice enhanced performance [S9, S10].

4) Activities of Daily Living (ADL) index (Modified Barthel’s Activities of Daily Living) [S11-S13] is completed by the patient/caretaker in a medical center environment in the presence of a health professional to provide a standardized environment for data collection. The original ADL was designed to evaluate the abilities and progress of patients with neuromuscular/musculoskeletal disorders to care independently for themselves in ten categories of basic self-care activities such as eating, dressing, grooming, personal toilet, and functional mobility. The score assigned was based upon the patient's actual, not potential, ability to perform specific activities. To make the ADL a more comprehensive assessment of ability to function beyond basic daily self-care activities, additional modules related to more complex and communal daily living activities (Supplementary Table S4) were added. Two components, not relevant to the CFS population (control of bowel and bladder function) were deleted. The overall ADL score is an average of all module scores and is a very internally consistent measurement of activity change [S11]. The average for the scores in 13 modules is multiplied by 20; since there are 5 levels of performance within the component modules. The maximum score is 100, which reflects an asymptomatic individual who can fully perform all eighty-three discrete activities of daily living.

5) Short Form 36 (SF-36) is a self-administered questionnaire (36 questions) that has been validated in the social science and medical literature and is used as a tool for assessing patient outcomes [S14,S15]. The two subscales used in this study were “vitality” (well-being, energy, fatigue) and “general health” (physical health status).

6) Activity Monitoring using a small, light-weight device (Computer Science and Applications, Inc.), which can be worn either on the wrist or on a belt fastened around the waist is a real time, objective measurement of physical activity. The device uses a piezoelectric crystal as the sensor to detect accelerations. The device then samples for accelerations ten times per second, and counts the number of accelerations within certain intervals, such as a three minute interval. It can then store the acceleration data in memory up to eight or nine weeks worth of data which can be downloaded for daily activity analysis.

7) Concomitant Medication usage is a reflection of the extensive medications used by CFS/ME patients to relieve symptoms of the disease. The use of concomitant medications as an efficacy endpoint in clinical trials evaluating the efficacy of drugs is discussed in a FDA Guidance document [S16]. This Guidance discusses the use of concomitant medications for pain relief (analgesic use) or as additional therapy for RA (arthritis treatment) and is applicable to CFS/ME.

8) Investigator Assessment of CFS Signs and Symptoms records whether patients experienced specific symptoms associated with CFS (Supplementary Table S6) within 4 week observation periods. Unlike the CFS-Related Symptom Score from the SCL-90-R, which captures the relative degree of CFS symptom-related impairment, the Investigator Assessment identifies whether or not the specific symptom occurred at all during the

intervening 30 day period. The Investigator Assessment does not quantify either symptom severity or the frequency of its occurrence within the 30 day time frame.

9) Hospital/Emergency Room Admissions and the number of hospitalized days is an objective measure of the quality of life for severely affected CFS/ME patients, which have been conducted but not publically reported to date [48].

C. Supplementary References

S1. Strayer DR, Carter W, Strauss KI, et al. Long term improvements in patients with Chronic Fatigue Syndrome treated with Ampligen. J Chronic Fatigue Syndrome 1995;1:35-53.

S2. Strayer DR, Carter WA, Brodsky I, et al. A controlled clinical trial with a specifically configured RNA drug, poly(I).poly(C12U), in chronic fatigue syndrome. Clin Infect Dis 1994;18 Suppl 1:S88-95.

S3. Current Hemispherx Biopharma NDA submission documents.

S4. Strayer DR, Carter WA, Stouch BC, et al. A double-blind, placebo-controlled, randomized, clinical trial of the TLR-3 agonist rintatolimod in severe cases of chronic fatigue syndrome. PLoS One. 2012;7(3):e31334. doi: 10.1371/journal.pone.0031334.

S5. Capewell S, Sudlow MF. Performance and prognosis in patients with lung cancer. The Edinburgh Lung Cancer Group. Thorax. 1990;45:951-6.

S6. McClellan WM, Anson C, Birkeli K, Tuttle E. Functional status and quality of life: predictors of early mortality among patients entering treatment for end stage renal disease. J Clin Epidemiol. 1991;44:83-9

S7. Wu AW, Mathews WC, Brysk LT, Atkinson JH, Grant I, Abramson I, Kennedy CJ, McCutchan JA, Spector SA, Richman DD. Quality of life in a placebo-controlled trial of zidovudine in patients with AIDS and AIDS-related complex. J Acquir Immune Defic Syndr. 1990;3:683-90.

S8 Snell CR, Stevens SR, Davenport TE, Van Ness JM. Discriminative validity of metabolic and workload measurements for identifying people with chronic fatigue syndrome. Phys Ther. 2013;93:1484-92.

S9. O'Donnell WE, DeSoto CB, Reynolds DM. A cognitive deficit subscale of the SCL-90-R. J Clin Psychol. 1984;40:241-6.

S10 Matarazzo JD, Weins AN, Matarazzo RG, Goldstein SG. Psychometric and clinical test- retest reliability of the Halstead impairment index in a sample of healthy, young, normal men. J Neuro Ment Dis. 1974;158-37-49.

S11. Mahoney FI, barthel DW. Functional Evaluation: THE BARTHEL INDEX. MD State Med J. 1965;14:61-5.

S12. Gresham GE, Phillips TF, Labi ML. ADL status in stroke: relative merits of three standard indexes. Arch Phys Med Rehabil. 1980;61:355-8.

S13. Collin C, Wade DT, Davies S, Horne V. The Barthel ADL Index: a reliability study. Int Disabil Stud. 1988;10:61-3.

S14. Ware JE, Sherbourne CD. The MOS 36-item short-form health survey (SF-36): I. Conceptual framework and item selection. Med Care. 1992;30:473-481.

S15. Ware JE, Snow KK, Kosinsiki M, Gendeck B. SF-36 Health Survey: Manual and interpretational Guide. Boston: The Health Institute, New England Medical Center, 1993.
S16. Guidance for Industry – Clinical Development Programs for Drugs, Devices and Biological Products for the Treatment of Rheumatoid Arthritis (RA) (February 1999), Section III E:16-30; Efficacy Trial Considerations.

D. Supplementary Tables

**Table S1. General Demographics of AMP-502 CFS/ME Patients**

| **Patient Demographics** | **Cohort Treatment Assignment** | | **Student t test** |
|---|---|---|---|
| | Rintatolimod | Placebo | |
| | 45 | 47 | |
| **Age** | **Years** | **Years** | |
| Average | 41.7 | 38.9 | p = 0.11 |
| Range | 25-69 | 25-53 | |
| Average Age at Diagnosis of CFS | 38.9 | 36.5 | p = 0.16 |
| Range | 22-53 | 22-52 | |
| Average Age at Onset of Symptoms | 35.6 | 34.5 | p = 0.57 |
| Range | (15.9 - 63.8) | (17.9 - 50.7) | |
| **Duration** | Years | Years | |
| Average Duration of Symptoms at Study Entry | 6.13 | 4.38 | p = 0.08 |
| Average Length of Illness (From Diagnosis to Study Entry) | 2.75 | 2.35 | p = 0.23 |

**Table S2 Karnofsky Performance Scale**

| | |
|---|---|
| 100 | Normal activity; no complaints; no evidence of disease. |
| 90 | Able to carry on normal activity; minor signs or symptoms of disease. |
| 80 | Normal activity with effort; some signs or symptoms of disease. |
| 70 | Cares for self, unable to carry on normal activity or do active work. |
| 60 | Requires occasional assistance but is able to care for most of needs. |
| 50 | Requires considerable assistance for daily care. |
| 40 | Disabled; unable to care for self, requires special care and assistance. |
| 30 | Severely disabled; bedridden although death is not imminent. |
| 20 | Very sick; hospitalization and/or nursing care is necessary; active support treatment is necessary. |
| 10 | Moribund; fatal processes progressing rapidly. |
| 0 | Dead. |

## Table S3. Treadmill Exercise Testing Protocol

| Stage | Minutes of Exercise (Elapsed) | Elevation % | Increment % | Belt Speed (MPH) | Increment (MPH) |
|---|---|---|---|---|---|
| I | 0 | 0 | - | 2 | - |
| II | 2 | 3 | 3 | 2 | 0 |
| III | 4 | 6 | 3 | 2 | 0 |
| IV | 6 | 9 | 3 | 2 | 0 |
| V | 8 | 12 | 3 | 2 | 0 |
| VI | 10 | 15 | 3 | 2 | 0 |
| VII | 12 | 18 | 3 | 2 | 0 |
| VIII | 14 | 21 | 3 | 2 | 0 |
| IX | 16 | 21 | 0 | 3 | 1 |
| X | 18 | 21 | 0 | 4 | 1 |
| XI | 20 | 21 | 0 | 5 | 1 |
| XII | 22 | 21 | 0 | 5 | 0 |

## Table S4. Cognitive Deficit Subscale Within the SCL-90-R Test

| **How much were you distressed by:** |
|---|
| Trouble remembering things |
| Having to do things very slowly to insure correctness |
| Trouble concentrating |
| Having to check and double-check what you do |
| Headaches |
| Temper outburst you could not control |
| The idea that something is wrong with your body |
| Your mind going blank |

| Table S5. ADL Activity Modules | | |
|---|---|---|
| Bathing | 8 | Meal Preparation |
| House Cleaning | 9 | Mobility |
| Communication | 10 | Physical Manipulation |
| Dressing | 11 | Transportation (Vehicular) |
| Grooming | 12 | Toilet |
| Home Management | 13 | Yardwork, Maintenance |
| Laundry | | |

| **Grading of ADL Tasks** |
|---|
| Unable to do |
| Need help most of the time |
| Need help some time |
| No help needed, symptoms present |
| No help needed, no symptoms |

| **Table S6. Investigator Assessment for Present or Absence of CFS Signs and Symptoms** |
|---|
| Mild Fever |
| Sore Throat |
| Painful Anterior or Posterior Cervical or Axillary Lymph Nodes |
| Unexplained generalized Muscle weakness |
| Muscle Discomfort or Myalgia |
| Prolonged (24 + hours) generalized fatigue |
| Migratory arthralgia without joint swelling or redness |
| Non exudative pharyngitis |
| Palpable or tender anterior or posterior cervical or axillary lymph nodes |
